# Diagnosis and management of diabetic neuropathy

**DOI:** 10.1186/1757-1146-4-S1-A2

**Published:** 2011-05-20

**Authors:** Andrew JM Boulton

**Affiliations:** 1 University of Manchester, Manchester Royal Infirmary, Oxford Road, Manchester, M13 PWL, UK; 2 University of Miami, Miami, FL, USA

## 

The diabetic neuropathies are common and chronic sensorimotor diabetic peripheral neuropathy (DPN) affects up to 50% of older type 2 diabetic patients. Up to half of these individuals may have painful symptoms of whom up to 20% will require some form of pharmacological therapy. The diagnosis of DPN remains a clinical one with exclusion of other causes of neuropathy important as no tests can determine that the neuropathy in any patient is caused by the diabetes. The most troublesome neuropathic symptoms include burning discomfort, altered temperature sensation (feet feel very hot or very cold), hyperaesthesiae, tingling, prickling and sudden shooting, stabbing pains. Examination usually reveals a stocking distribution sensory loss although in acute sensory neuropathy, the clinical examination may be normal. Evidence suggests that blood glucose flux, with erratic fluctuations of blood glucose during the day and night, contributes to the pathogenesis of neuropathic pain. With respect to treatments, the first step is to try and achieve optimal, stable glycaemic control: sudden improvement in control may however actually worsen neuropathic symptomatology. In addition to achieving stable glycaemic control, most patients require some form of pharmacological intervention and first line drugs include anti-epileptics such as Gabapentin and Pregabalin, the antidepressant and dual reuptake inhibitor, Duloxetine, or the tricyclic drug Amitriptyline. Strong evidence from randomised controlled trials supports the use of each of the above agents. For those patients with resistant neuropathic pain, synthetic Opioids such as Tramadol, or stronger drugs such as slow release Oxycodone may be useful in the short term. To date, no specific pathogenetic treatments are licensed in the UK for the management of neuropathic pain.

